# Inferring polydomy: a review of functional, spatial and genetic methods for identifying colony boundaries

**DOI:** 10.1007/s00040-016-0534-7

**Published:** 2016-12-05

**Authors:** S. Ellis, D. S. Procter, P. Buckham-Bonnett, E. J. H. Robinson

**Affiliations:** 1 Department of Biology and York Centre for Complex Systems Analysis, University of York, York, UK; 2 Centre for Research in Animal Behaviour, University of Exeter, Exeter, UK; 3 Centre for Exercise, Nutrition and Health Sciences, School of Policy Studies, University of Bristol, Bristol, UK

**Keywords:** Polydomy, Colony boundaries, Social organisation, Genetic differentiation, Spatial clustering, Resource movement

## Abstract

Identifying the boundaries of a social insect colony is vital for properly understanding its ecological function and evolution. Many species of ants are polydomous: colonies inhabit multiple, spatially separated, nests. Ascertaining which nests are parts of the same colony is an important consideration when studying polydomous populations. In this paper, we review the methods that are used to identify which nests are parts of the same polydomous colony and to determine the boundaries of colonies. Specifically, we define and discuss three broad categories of approach: identifying nests sharing resources, identifying nests sharing space, and identifying nests sharing genes. For each of these approaches, we review the theoretical basis, the limitations of the approach and the methods that can be used to implement it. We argue that all three broad approaches have merits and weaknesses, and provide a methodological comparison to help researchers select the tool appropriate for the biological question they are investigating.

## Introduction

Social insect colonies are often viewed as a ‘factory within a fortress’, living colonially to produce and defend the next generation (Wilson 1971). Within this ‘fortress’, the insects share resources, risks, and reproductive effort. To study social insects properly it is, therefore, important to clearly determine the boundaries of ‘a colony’. The position of colony boundaries has a range of impacts on the evolution and ecology of ants, and is an important consideration for experimental design.

From an evolutionary perspective, determining the boundaries of a colony is vital for understanding and studying how selection is acting within a population of social insect colonies. In eusocial insect colonies, selection can act at multiple levels including between the individuals within a colony, and between separate colonies (Bourke and Franks 1995). Clearly, colony-level selection cannot be understood without identifying the boundaries of the colonies in question. Similarly, for within-colony selection, knowing which individuals are part of the same colony is necessary to understand the selective forces acting within the system. The fitness of an individual or colony depends on its ecology, its relationship with other organisms and on the environment. In social insects, ‘population’ can refer to the number of colonies present or to the number of individual insects present, both of which are likely to have important impacts on the wider ecosystem. To accurately assess the number of colonies present in a population, it is again necessary to correctly determine the colony boundaries. Appreciating the role of colonies (rather than just number of individuals) within an ecosystem is particularly important when considering species conservation, and the impacts of invasive species. The level of urgency of conservation efforts, and the optimal conservation strategy will depend on the population size of the species in question (Shaffer 1981). Similarly, the impacts of an invasive species, and the likelihood of eradication attempts being successful, will depend on the population size of that species (Hoffmann et al. 2016). Finally, when planning experiments that use social insects, determining colony boundaries is vital for ensuring the generation of scientifically robust conclusions. Fair sampling and adequate repetition are an important part of the scientific process and the repeats and samples should, as far as possible, be independent of each other. In social insects, this means that it is important to repeat an experiment in different colonies. It is clear that the correct identification of boundaries is essential for the identification of colonies, and therefore the facilitation of proper experimental design.

Ant colonies have been traditionally viewed as a collection of closely related females living in a single nest, producing and defending the next generation (e.g. Wilson 1971). In recent decades, this view has been found to be an underestimation of the social complexity of colonies in most ant species (Heinze 2008). A particularly striking example of this complexity is the distribution of the colonies of some ant species across multiple nests, a strategy called polydomy. Polydomy has evolved many times independently in ants; it is found in at least 166 species, and is likely to be present in more (Debout et al. 2007). Identifying the boundary of a polydomous colony can be challenging. Rather than simply being able to identify individual nests, it is necessary to determine which groups of nests within the population are parts of the same multi-nest colonies.

An important first step for identifying the boundaries of a multi-nest colony is to clearly define the meaning of polydomy. The most generally accepted definition of polydomy is a colony inhabiting several spatially separated (by greater than the usual distance between nest chambers) but socially connected nests (Debout et al. 2007; Robinson 2014). A nest, for the purpose of this definition, is defined as any structure containing both workers and brood (Debout et al. 2007). ‘Colony’ and ‘social connection’ are more challenging to define. The term colony is used broadly by Wilson (1971) to mean a society of ants or other social insects; more restrictive definitions have included the sharing of resources and reproduction (Gordon and Heller 2012) and the ability to distinguish group members from outsiders and reject outsiders on that basis (Moffett 2012a). We aim to inform the discussion of colony boundaries by examining the assumptions and implications of various approaches which have been used to delineate colony boundaries. Each approach implicitly uses a particular restrictive definition of colony, and we will discuss the ecological and evolutionary limitations of these restrictive definitions. It is important to note that the boundaries of a colony are not necessarily static and may change with time. For example, many species show seasonal changes in colony structure, founding new nests at certain times of year, and abandoning them at others (e.g. Banschbach and Herbers 1996a; Heller and Gordon 2006; Buczkowski and Bennett 2008). By defining a colony by the boundary of its social connections, this dynamism can be incorporated. To identify whether nests are part of the same polydomous colony, it is therefore necessary to determine what is meant by a ‘social connection’ between nests, and to then assess whether such a connections exists.

**Fig. 1:**
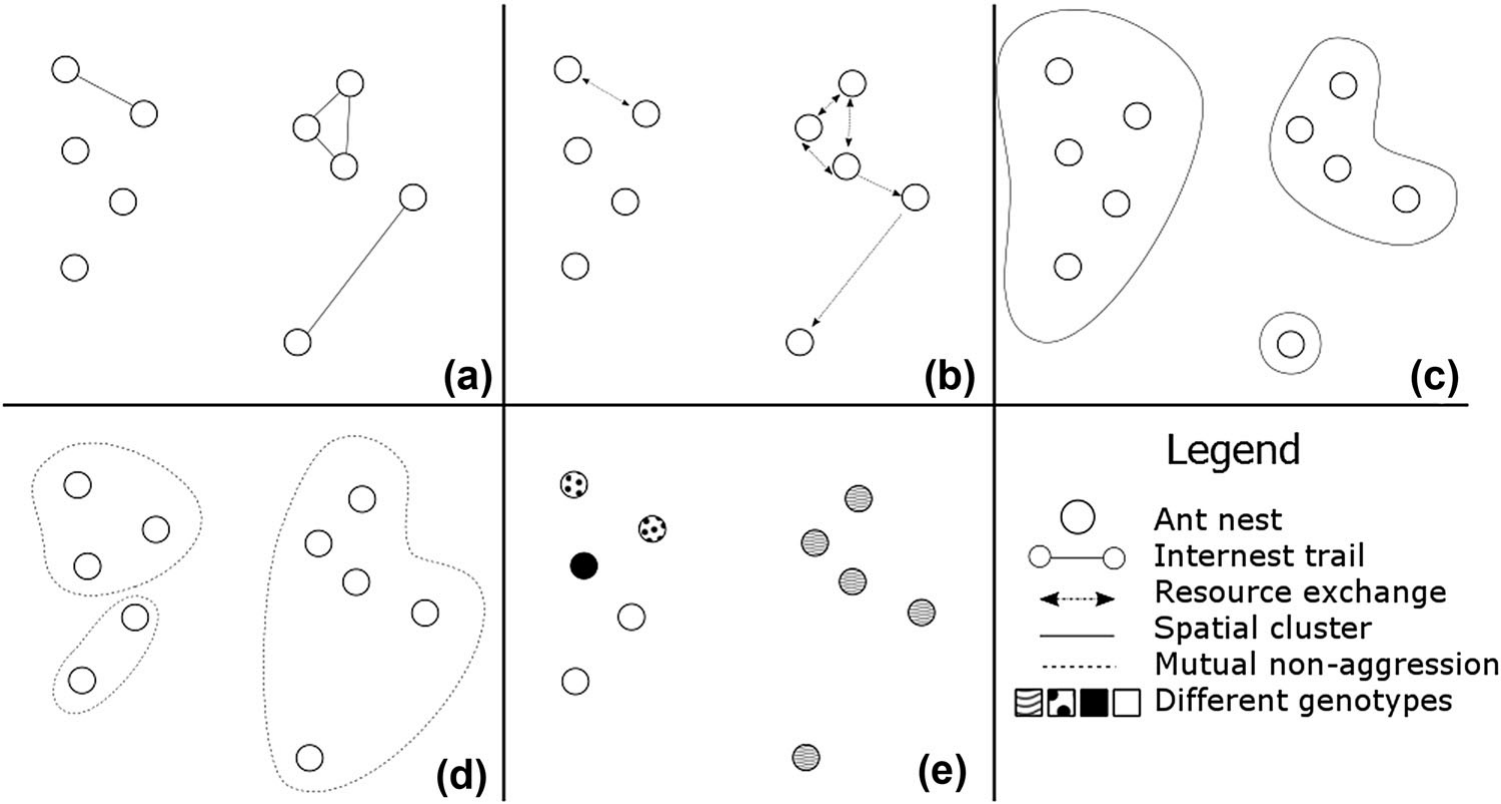
A hypothetical set of ant nests (*circles*) and the relationships between them drawn by different methods of polydomous colony delineation. **a** Worker trails, denoted by *lines* between nests; **b** resource exchange, denoted by *dotted lines* with *arrows* showing the direction of resource movement; **c** Spatial clustering, denoted by *lines* around clusters; **d** mutual non-aggression, denoted by *dotted lines* around groups; **e** different genetic groupings, denoted by patterns within the *circles*. Real polydomous colonies are likely to show much lower variation between different methods; we vary the results to demonstrate different methods may not agree

A variety of approaches have been used to determine whether there is a social connection between nests, which can be broadly classified as those based on nests: (i) sharing resources (both environmentally derived resources and the colony members themselves), (ii) sharing space, and (iii) sharing genes (Table 1; Fig. 1). These approaches utilise a range of methods. In this paper, we review the approaches and methods for delineating colony boundaries in polydomous species and evaluate their aims, implications, assumptions, advantages and disadvantages.

**Table 1:** A summary of methods for identifying the boundaries of polydomous colonies, their utility and limitations, and the knowledge required to use them

Method	Utility (example references)	Limitations	Prerequisite knowledge	Potential biases
Marked food (e.g. immunoglobulin marking)	Movement of marked resources shows that ants from those nests are moving between nests or exchanging food (Buczkowski and Bennett 2009; Hoffmann 2014)	Function of resource sharing (e.g. sharing, stealing, and appeasement) is unknown. There are also sampling difficulties, especially within populous nests	The type of resources being exchanged between nests. Ideally, also the method by which the exchange takes place and the level of resource exchange between a pair of nests	Underestimation of colony size due to: – insufficient resource marking – heterogeneous resource movement Overestimation of colony size due to: – stealing of food by neighbours
Marked workers	By marking workers and observing their movement, the behaviours associated with a social connection can be studied. If resource exchange occurs, information about the mechanism will be revealed (Rosengren 1985; Ellis and Robinson 2016)	The re-observation rate of marked workers (and therefore the number of workers that need to be marked) will depend on the population of the nests in question and the complexity of the system of behaviours involved in inter-nest resource transfer. Marked workers in nests with large populations or complex resource exchange mechanisms are unlikely to be re-observed	Durability of individual markings Probability of re-observation of a marked individual over a time-period relevant to the study, to determine required number of marked individuals	Underestimation of colony size due to: – marking an insufficient number of workers – loss of markings – heterogeneous worker movement – high nest fidelity
Direct observation of trails between nests	Gives a good quantitative overview of the structure of social connections over a whole multi-nest system; can provide quantitative data about connection strengths from trail usage (van Wilgenburg and Elgar 2007; Ellis et al. 2014)	The nature of resources being exchanged via trails is unclear; trail usage may not be a good approximation of resource exchange via trails; mechanism of exchange is unknown	Only appropriate if the species consistently forms trails between all nests that exchange resources	Underestimation of colony size due to: – failing to observe trails that are used inconsistently – failing to record underground connections
Ecological inference (e.g. changed nest strategy in response to environmental change)	Puts the social connection, and potentially resource exchange, between nests in a clear ecological context (Banschbach and Herbers 1996a; Dahbi et al. 2008)	The nature and extent of resource exchange, the quantities exchanged and the mechanism of exchange are unclear. The timescale (i.e. temporary or long-term) of the strategy are also unknown	That observed changes in the nesting strategy are not simply a short-term intermediate strategy, rather part of a long-term, and evolutionarily relevant, strategy	Misidentification of colony boundaries due to: – observer bias – inaccurate identification of the cause of nest separation
Inter-nest aggression assays	Demonstrates whether workers from a pair of nests are mutually tolerant, or mutually aggressive (Roulston et al. 2003)	There are a great variety of types of assays which can, and have, been used to investigate aggression between nests (Table 2). The efficacy and consistency of these various methods are unknown. Observer bias is problematic when subjectively identifying aggression between ants. Some species of ants are non-aggressive, even to conspecifics from distant populations	That aggression is expected between ants from different colonies, and that this aggression will be reproduced consistently in the assay being used	Overestimation of colony size due to: – low overall aggression in population/species – low motivation for aggression due to, e.g. season or context – observer bias Underestimation of colony size due to: – inappropriate testing conditions causing increased aggression – observer bias
Spatial clustering analysis	An objective technique to assess whether nests are distributed non-randomly in the environment (Sudd et al. 1977; Santini et al. 2011)	Both ecological factors and population history can produce clusters of nests in the environment. The scale at which clustering is investigated is also subjective. Methodological difficulties with defining the boundaries of the area in which clustering is to be assessed	The impact of environmental limitations on space occupancy, so that this effect can be distinguished from the effects of space sharing	Over-or underestimation of colony size due to: – failing to identify an important environmental variable
Genetic differentiation, *F*_ST_	Workers displaying significant genetic differentiation are unlikely to be within the same reproductive unit, and therefore the same colony (Elias et al. 2004; Steinmeyer et al. 2012)	Differentiation builds up over long time scales, potentially longer than colony formation, therefore lack of differentiation does not mean that two nests are within the same colony	Any evidence that colony formation is likely to be very recent, such as recent population expansions. Is genetic differentiation detectable in the population as a whole?	Overestimation of colony size due to low differentiation, caused by: – recent founding – low power
Relatedness	Highly related workers are very likely to be from the same family unit, and therefore the same colony (Pedersen and Boomsma 1999; Pamminger et al. 2014)	In highly polygynous populations, relatedness can be indistinguishable from zero. Relatedness estimates are also highly variable within a nest, therefore it may be difficult to distinguish between nests showing small differences in relatedness	The expected level of polygyny within the population	Overestimation of colony size due to: – high variability causing lack of ability to distinguish nests on relatedness
G-distance	A comparative measure of differentiation, G-distance describes how genetically different workers are (Pedersen and Boomsma 1999)	It is impossible to compare different studies, because measures are comparative within studies. There is no obvious cut off above which colony boundaries are clear		Underestimation of colony size due to: – assumptions that there are distinctions within a population, and that comparative measure will be useful
Rare genotype sisterhoods	Nests sharing rare genotype sisterhoods share common descent, which can reveal groupings within highly variable data (Pedersen and Boomsma 1999)	Only works if there are sufficiently rare alleles. Not identifying a sisterhood does not mean that two nests are within different colonies. In recently expanded populations, many different colonies may share descent and therefore share rare genotype sisterhoods	Evidence of recent population formation or bottlenecks: if present these may obscure rare alleles, because all members of population share recent descent	Overestimation of colony size due to: – population lacking sufficiently rare genotypes – recent population expansion
Bayesian clustering methods	Bayesian clustering methods allow delineation of genetic groupings without observer bias (Holzer et al. 2009; Huszár et al. 2014)	Any genetic structure in the data will be identified, not necessarily colony boundaries, e.g. a population formed by the merging of two distinct gene pools may separate by those gene pools, even though each contains many colonies	Any genetic structuring within the population that is not related to colony structure, e.g. differentiation due to a geographic barrier	Overestimation of colony size due to: – genetic groupings above the colony level being misidentified as colony boundaries Underestimation of colony size due to – genetic isolation by distance within large polydomous colonies being misinterpreted as colony boundaries
Sequencing mtDNA	mtDNA haplotypes shared between nests is evidence of shared descent (Holzer et al. 2009; Seppä et al. 2012)	Variability can be low across large areas; there may not be mtDNA variation within the population at all	Variability of mtDNA within population or region	Overestimation of colony size due to: – lack of variation within populations Underestimation of colony size due to: – multiple haplotypes within a colony leading to incorrect inference of a division

Examples of studies are included in the ‘utility’ column, but for more complete referencing refer to the text

## Sharing resources

Sharing resources is an important aspect of sociality in a wide range of organisms (Krause et al. 2015). In social insects, resource sharing is a fundamental feature of eusociality, and shapes colony structure (Oster and Wilson 1978). The sharing of resources between nests can therefore be a useful, and biologically relevant, way to define a social connection between nests because it extends a pre-existing within-nest process (resource sharing) to the interactions between nests. The sharing of resources between nests suggests that those nests are members of a single cooperative unit, a colony.

There are two broad categories of resources which nests could share: resources derived from the environment and the ants themselves (i.e. reproductives, brood and workers). Conceptually, these categories of resource are very different, but the processes by which they are shared are interconnected. Environmentally derived resources are those collected by workers from the area around a nest, for example food or nesting materials. In a monodomous colony, resources collected from the environment are transported to the single nest, which can therefore be thought of a single functional unit with regards to its interactions with the environment. If multiple nests are sharing resources, they can similarly be thought of as a single functional unit when examining their interactions with the environment. For example, if multiple nests share food, they are pooling their foraging effort, meaning that their interactions with the environment are interrelated. As the interactions are not independent, the multi-nest system can be considered a functional unit.

Reproductive individuals, brood and workers, can be viewed as resource that can be shared between nests. Division of reproductive effort is a defining trait of eusocial societies (Oster and Wilson 1978; Bourke and Franks 1995), and therefore the sharing of reproductive effort between nests would be an important indicator of nests being part of the same society. In a monodomous colony, all reproductive individuals and brood are tended and protected in the same nest, meaning that the brood share the same developmental environment and have linked fitness prospects (Bourke and Franks 1995). Similarly, in a polydomous system, reproductive individuals, brood and workers moving between nests will lead to ants in these nests sharing fitness prospects.

These potential social connections between nests, implied by the sharing of either environmentally derived resources or of colony members, are closely linked. For example, environmentally derived resources shared between nests are likely to be used to support the raising of brood, which will result in the fitness prospects of the brood in different nests becoming linked. Similarly, brood transported between nests may develop into workers which will then aid their receiving nest in collecting resources from the environment. The extent to which nests sharing resources can be considered a single ecological or evolutionary unit will depend on the types of resources being exchanged, the level of exchange, and the life-history of the species being studied.

### Limitations

Not all resource transfer between nests is due to cooperation. For example, some monodomous species have been observed stealing food (Breed et al. 1990; Yamada 1995) or brood (Pollock and Rissing 1989) from neighbouring conspecific colonies, a behaviour called intraspecific kleptoparasitism. Whilst during kleptoparasitism resources are transferred from one nest to another, it is not an example of resource sharing; the resources are being taken at a cost to the targeted nest, without the benefits accrued from a shared genetic heritage. Sharing, as opposed to stealing, requires the cooperation of the nests from which the resources are being taken. Sufficient knowledge of the life-history of a species and observation of the interactions between workers are needed to establish if resources are truly being cooperatively transferred between nests, rather than simply stolen.

Theft and sharing are not the only options. Resources are sometimes exchanged between individuals from different nests as part of appeasement behaviours. Workers from ecologically subordinate ant species have been observed surrendering food to the ecologically dominant and invasive species *Solenopsis invicta* (Bhatkar and Kloft 1977). Exchanging resources appears to appease *S. invicta* workers, reducing aggression and perhaps giving the donor time to escape (Bhatkar and Kloft 1977). Appeasement may also occur intraspecifically, for example, *Formica paralugubris* workers give food to workers from other nests significantly more often than workers from their own nest (Chapuisat et al. 2005). The extent to which intraspecific appeasement can be considered synonymous with sharing resources, and therefore membership of the same multi-nest colony will depend on the behavioural and ecological conditions under which the exchange takes place. Further research is necessary to establish the extent of intraspecific appeasement in ants, and how it differs behaviourally from resource sharing.

For a worker ant, the ability to correctly identify other workers who are members of the same colony is important to ensure that resources are shared in a way that enhances its inclusive fitness. Mistakes in recognition could result in workers exchanging resources with workers from nests which are not part of the same colony. It has been suggested that polydomy may simply result from the failure of workers to differentiate between nestmates and non-nestmates when transferring resources (Tsutsui et al. 2000, 2003; Chapman and Bourke 2001; Giraud et al. 2002). However, more recent work has suggested that workers from different nests can recognise other workers as nestmates or non-nestmates even in the absence of an aggressive reaction (Holzer et al. 2006), in the presence of resource exchange (Chapuisat et al. 2005) or with other confounding factors such as high intra-nest genetic diversity (Helanterä et al. 2011). Even if recognition-failure is the proximate mechanism of resource sharing within a multi-nest system, it will still result in resources being exchanged between several nests. This makes those nests a single functional unit with regards to their interactions with the environment, and therefore potentially a unit of selection. Inability to discriminate, therefore, does not necessarily mean that defining the boundary of a colony using resource sharing is an invalid approach; the ants’ lack of discrimination may simply be the mechanism by which the system is maintained.

### Methods

The limitations of using resource sharing as an approach for delimiting colony boundaries can be overcome, or at least controlled for, by good experimental design. A range of methods have been used to find the resource exchange boundaries of a colony. These methods are often based on either tracking resource movement between nests, tracking the workers transporting the resources, or inferred from the relationship between the resource environment and ant life-history and behaviour (Table 1).

Marking resources, and then observing their passage through the population, can be a useful way to directly trace functional social connections between nests. For environmentally derived resources, this usually involves placing a marked resource, often food, in the vicinity of a given nest (Buczkowski and Bennett 2009; Buczkowski 2012; Hoffmann 2014; Procter et al. 2016). Later, the presence or absence of the marked resource in other nests in the population can be tested. Finding the marked resource in another nest suggests that the resource has been distributed from the nest near the food to the others in the population, i.e. shared. This implies membership of the same resource sharing polydomous colony.

The advantage of using marked resources to examine social connections between nests is that the presence of a marked resource in non-source nests is direct evidence that resources are being moved between those nests, at least in one direction. However, although movement is directly measured, the nature of the exchange is not assessed. This limitation means that marked resource methods cannot distinguish between resource sharing, appeasement and intraspecific kleptoparasitism. Resource marking has been used successfully to observe resource exchange between nests in some species (Buczkowski 2012; Hoffmann 2014; Procter et al. 2016), however, this method becomes more challenging and may be less informative in species with very large worker populations. Marked resources may be difficult to detect if they are masked by the large quantities of unmarked resources being collected. To conclude with confidence that there is an absence of resource exchange between nests in a large population would require very extensive sampling, which may be impractical. The use of the resource marking methods can be successful, but this success will depend on the ecology and life-history of the species being studied.

In a mature colony, all resources must ultimately be transported by workers, either internally or in the mandibles. Workers transporting resources between nests can be marked and their visiting behaviour directly observed (O’Neill 1988; Ellis and Robinson 2015, 2016). Observing the movement of workers between nests is arguably the most direct way of observing a resource sharing social connection between nests. Marking workers, usually with paint, has given important insights into the redistribution of resources within and between nests (e.g. Rosengren 1985; Ellis and Robinson 2016). With technological advances (such as RFID tags, e.g. Robinson et al. 2009) more detailed study of the movement of resources within ant colonies is now possible. Workers themselves can also be a considered a resource, so tracking worker movement can be beneficial even in the absence of obvious transportation of environmentally derived resources. Genetic methods are usually used to track the movement of reproductive individuals, brood and workers between nests, and this is discussed in the ‘sharing genes’ section of this review (below). Direct observation of resource transport has the advantage of establishing that resources are definitely being shared between the nests in question, and are not being exchanged via appeasement behaviours. The disadvantage of direct observation and marking is that it can be time consuming and labour intensive, especially for species that have many nests in their polydomous colonies.

By making the assumption that workers visiting other nests are transporting resources, without detailed marking and tracking of the workers, some of the time and labour disadvantages of directly marking resources can be overcome. In some species, observing worker travel between nests is simplified because workers move along clearly defined trails between nests. For example, polydomous colonies of red wood ants (*Formica rufa* group) form strong trails between nests, often consisting of thousands of ants (Ellis and Robinson 2014). Identifying the resource sharing boundaries of a red wood ant colony is therefore a matter of following the trails between nests to determine connections (e.g. Cherix 1980; Ellis et al. 2014; Ellis and Robinson 2015; Ellis and Robinson 2016; Procter et al. 2016). Species with long-lasting and strong trails between nests tend to be those whose nests are themselves long-lived and populous, such as *Camponotus gigas* (Pfeiffer and Linsenmair 1998), *Iridomyrmex* spp. (McIver 1991; van Wilgenburg and Elgar 2007) and *Myrmicaria opaciventris* (Kenne and Dejean 1999). Some ants, including many invasive species, with a large number of less populous and ephemeral nests also link nests with trails (Vargo and Porter 1989; Heller and Gordon 2006). However, as the nests are more transient, the inter-nest trail structure is similarly less permanent and more subject to short-term changes (Heller and Gordon 2006). Mapping trails between nests has the advantage of establishing a direct social connection between nests using a relatively simple and time-efficient method, but maps must be interpreted in the light of the species’ ecology and environment.

Not all polydomous ant species use trails, and even trail-forming species may also use other, less obvious, forms of resource sharing between nests. For example in *Formica yessensis*, not all between nest trips follow fixed trails (Higashi 1978). An absence of visible connections may also be due to the inter-nest connections being underground. *Solenopsis invicta,* for example, can spread out to occupy multiple nests from a single origin without forming above-ground trails (Vargo and Porter 1989), whilst the above-ground inter-nest trails of *Myrmicaria opaciventris* finally become trenches, and then tunnels (Kenne and Dejean 1999). The disadvantage of using trails to infer social connection is that the efficacy of this method depends on the ecology of the species being studied. A lack of such species-specific knowledge may result in resource sharing connections between nests being missed, and therefore the true extent of a colony being underestimated.

The boundaries of a polydomous colony can sometimes be inferred indirectly by observing how the colony system reacts to environmental changes. For example, *Crematogaster torosa* and *Linepithema humile* found new nests close to new sources of food, suggesting a link between resource collection and polydomy (Holway and Case 2000; Lanan et al. 2011). Similarly, food and nest-site limitations have been linked to seasonal polydomy in several ant species, suggesting that polydomy is a response to a lack of resources in the environment (Banschbach and Herbers 1996a; Heller and Gordon 2006; Buczkowski and Bennett 2008). The transport of workers and brood can also be induced by changes in the environment. When *Cataglyphis iberica* nests are attacked, workers and brood are transported from the attacked nests to other neighbouring nests (Dahbi et al. 2008). The movement of resources between nests in response to changes in the environment clearly shows that a social connection exists between the nests in question. However, the longevity of these changes is often unclear. The occupation of multiple nests could be part of a long-term strategy, but it could also be a short-term response to stressful conditions, with colonies reverting to a system lacking the social connection between nests after the triggering event. For example, some anecdotal evidence suggests that temporary polydomy can be a response to deteriorating environmental conditions in some members of the *F. rufa* group (Breen 1979; Sorvari and Hakkarainen 2005; Robinson and Robinson 2008). To correctly infer the effects of environmental change on resource sharing behaviour, and to distinguish between general nesting strategy and stress-related changes, long-term observations are needed.

Overall, measuring resource sharing can be a useful approach with which to infer a social connection between nests. The sharing of resources demonstrates that the nests are, in a sense, a single ecological unit (i.e. the nests’ interaction with the environment is interdependent). The connection between a functional, ecological unit and a unit of selection is unclear and will depend on the type of resources being shared between nests, and the ecology and life-history of the species being studied. The methods used to investigate resource sharing between nests are, in general, fairly simple and inexpensive. However, they require good species knowledge to demonstrate that the assumptions of the methods being used are valid.

## Sharing space

The absence of aggression between nests and a clustering of nests in the environment are both criteria which are commonly used to infer membership of a polydomous colony. Though it is rarely explicitly stated, these approaches imply a measure of social connection based on a shared space. If members of a pair of nests are not aggressive towards each other, then it may imply that they are willing to share their foraging area (even if the nests are too far away for this to actually occur); indirect resource sharing; and perhaps shared descent. Together, these consequences of sharing a space may suggest that the nests can be considered part of the same colony. Similarly, if nests are clustered in space, it may imply a shared foraging area, and therefore mutual tolerance, meaning the nests could be considered part of the same colony. A measure of social connection based on a shared space suggests that the social connection between nests is based on a shared resource collection area, which may be indicative of some mutual aid, indirect resource sharing, and again, perhaps shared descent. In this section, we discuss how shared-space approaches to defining colony boundaries are used, their advantages, and their limitations.

### Aggression

Using aggression to define the boundaries of a multi-nest colony is based on the assumption that the lack of aggression between nests implies membership of a single cooperative unit (Moffett 2012; Kennedy et al. 2014). Inter-colony aggression is common in social insects. Intraspecific inter-colony aggression is often aimed at excluding other colonies from resources. Recognition of, and aggression towards individuals from other colonies is mediated by hydrocarbon compounds associated with the cuticle of the ants. These hydrocarbons are derived from both genetic and environmental sources (Martin and Drijfhout 2009). The extent to which the genetic and environmental factors influence hydrocarbon profiles varies between species (Buczkowski and Silverman 2006; Sorvari et al. 2008; Van Zweden et al. 2009). Broadly, all workers of the colony will share a similar cuticular hydrocarbon profile, and this facilitates recognition of, and aggression towards, non-nestmates. Assessing the boundary of a polydomous colony based on aggression aims to use the reaction of ants to workers from other nests to test whether their nests are part of the same colony.

### Limitations

Measuring aggression to determine colony boundaries has raised some problems. An important difficulty is that aggression carries costs. The potential for the loss of resources and workers means that the costs of aggression are high for ant colonies (Helanterä 2009). For aggression between individual ants to bring a fitness benefit to a colony, the costs of the behaviours must be outweighed by the results of being aggressive (Helanterä 2009). Therefore, the decision for workers from different colonies to act aggressively towards each other relies not only on them recognising their different colony odours, but also on their having sufficient motivation to act aggressively. Variation in the motivation of workers may confound the observation of aggression between individuals, and therefore frustrate efforts to define colony boundaries.

Resource limitation can be an important motivation for aggressive behaviours. In some ant species found in temperate regions, this is particularly evident in the seasonal variation in aggression. A variety of ant species have been observed to exhibit much higher aggression in spring than summer (Mabelis 1984; Katzerke et al. 2006; Thurin and Aron 2008). In spring, resources are likely to be limited, and therefore the benefits gained by monopolising a food source are high, making it beneficial to be more aggressive. Conversely in summer, resources are more abundant, and therefore aggression is not as beneficial. In species where aggressive responses vary with season, using aggression between nests to delimit a polydomous colony would result in different nests being considered as part of the same colony at some times of year, but as members of different colonies at others.

The importance of worker motivation to act aggressively is highlighted by the examples of some ant species in which, despite recognising non-nestmates, workers are not aggressive towards them (Greenslade and Halliday 1983; Holzer et al. 2006; Björkman-Chiswell et al. 2008). This can extend over large geographical areas. For example, different populations of *Formica paralugubris* in Switzerland show no aggression despite being widely separated and geographically distinct, but workers do show longer bouts of antennation towards workers from different populations (Holzer et al. 2006). This disjuncture between recognition and aggression has the potential to make defining colony boundaries by aggression difficult, because without species-specific knowledge, it is difficult to assess whether a lack of aggression truly indicates that workers are part of the same cooperating colony, or simply have no motivation to be aggressive.

The costs of aggression are also important for the ‘dear-enemy’ phenomenon (Ydenberg et al. 1988). The dear-enemy phenomenon describes how in many territorial species, including ants, individuals are less aggressive to neighbours than to strangers (Ydenberg et al. 1988). This has the potential to confound the assessment of colony boundaries using aggression. The dear-enemy phenomenon is likely to arise from the costs of aggression and the fact that once a territory boundary is recognised by both parties, it is counter-productive to continue to fight over it (Ydenberg et al. 1988). Strangers (i.e. not neighbours) are less likely to recognise the territory boundaries, and therefore pose more of a threat, eliciting an aggressive reaction (Ydenberg et al. 1988). This phenomenon has been found in a wide variety of ant species (Debout et al. 2003; van Wilgenburg 2007; Tanner and Keller 2012). The dear-enemy phenomenon poses problems for aggression-based measures of social connection between nests, because a lack of aggression in this case does not even represent shared foraging territory, simply a well-recognised boundary. However, it is also important to note that this is not universal, and in some species there is no relationship between spatial distance and level of aggression (Tanner and Keller 2012) and that other factors can influence aggression to neighbours such as familiarity and relatedness (van Wilgenburg 2007; Tanner and Keller 2012).

Seasonal variation in aggression and the dear-enemy phenomenon are both examples of how aggressive behaviours can be affected by the motivation of the individuals involved. A variety of other factors have been shown to influence the motivation of ants to act aggressively. These include the physical environment (Bengston and Dornhaus 2014), the social environment (Sakata and Katayama 2001), individual variation in worker aggression (Newey et al. 2010), individual experience (Signorotti et al. 2014), the inferred proximity of the home nest (Buczkowski and Silverman 2005) or the state of the opponent (Fortelius et al. 1993). The variation in aggression levels caused by factors affecting motivation is likely to confound efforts to use aggression as a tool to define colony boundaries.

### Methods

Testing for aggression between ants from different nests generally involves placing an ant (or ants) from one nest with an ant (or ants) from another nest and scoring the hostility of their interactions. If aggressive behaviours are robustly observed, the ants are considered to have originated in different colonies, and if not, they are considered part of the same colony. However, as discussed above, colony membership is not the only factor influencing whether workers show an aggressive response. When performing aggression assays, variation in the motivation of individuals to be aggressive can arise, not only from natural differences in individual motivation but also from experimentally introduced difference in motivation. There are a wide variety of methods which have been used to test for aggression between ants, varying in, for example, the numbers of ants used and the location of the tests (summarised in Table 2). Although most aggression assay methods show correlated results, the use of some methods is significantly more likely to result in the observation of aggression than other methods (Roulston et al. 2003). In addition, some methods produce a more repeatable aggressive response in individual ants than others (Roulston et al. 2003). The type of assay used can therefore affect the level of aggression of observed, and consequently the designation of colony boundaries within a population. It is beyond the scope of this review to evaluate the diversity of methods for assessing aggression between nests. Rather, we aim to highlight the fact that different methods can yield different results, and the choice of an appropriate test is imperative when assessing colony boundaries (Table 2).

**Table 2:** A summary of methods used to test aggression between ants

	Artificial conditions		Natural conditions
	Lab	Field	
Single vs. single	*Aphaenogaster senilis* [1] *Formica aquilonia* [2] *Formica paralugubris* [3] *Lasius neglectus* [4] *Oecophylla smaragdina* [5]	*Atta laevigata* [6]* *Camponotus pennsylvanicus* [7] *Crematogaster opuntiae* [8] *Lasius neoniger* [9] *Leptothorax cutteri* [10] *Linepithema humile* [11] *Plectroctena mandibularis* [12] *Streblognathus peetersi* [12]	
Single vs. group	*Ectatomma tuberculatum* [13]* *Nylanderia flavipes* [14] *Plagiolepis pygmaea* [15] *Oecophylla smaragdina* [16]	*Lasius neoniger* [17]	
Single vs. nest	*Linepithema humile* [18] *Myrmica punctiventris* [19] *Temnothorax rugulatus* [20]		*Cataulacus mckeyi* [21] *Ectatomma ruidum* [22]* *Rhytidoponera* sp. [23]
Group vs. group	*Oecophylla smaragdina* [5] *Linepithema humile* [25]	*Crematogaster scutellaris* [24] *Formica aquilonia* [26] *Iridomyrmex purpureus* [27]*	
Group vs. nest			*Ectatomma ruidum* [22]* *Formica exsecta* [28] *Formica pratensis* [29]
Nest vs. nest	*Anoplolepis gracilipes* [30]		*Solenopsis invicta* [31]

The list is not exhaustive but intends to give a summary of the diversity of the tests used. Lab and Field arenas refer to laboratory-based experiments and experiment performed in situ but with partially controlled conditions. References: 1. (Signorotti et al. 2014), 2. (Sorvari et al. 2008), 3. (Holzer et al. 2006), 4. (Ugelvig et al. 2008), 5. (Newey et al. 2010), 6. (Hernández et al. 2002), 7. (Buczkowski 2011), 8. (Lanan and Bronstein 2013), 9. (Buczkowski 2012), 10. (Allies et al. 1986), 11. (Vogel et al. 2009), 12. (Tanner and Keller 2012), 13. (Fénéron 1996), 14. (Ichinose 1991), 15. (Thurin and Aron 2008), 16. (Newey et al. 2008), 17. (Traniello and Levings 1986), 18. (Buczkowski and Silverman 2006), 19. (Banschbach and Herbers 1996a), 20. (Bengston and Dornhaus 2014), 21. (Debout et al. 2003), 22. (Breed et al. 1992), 23. (Pamilo et al. 1985), 24. (Santini et al. 2011), 27. (Björkman-Chiswell et al. 2008), 26. (Sorvari and Hakkarainen 2004), 27. (van Wilgenburg 2007), 28. (Katzerke et al. 2006), 29. (Pirk et al. 2001), 30. (Hoffmann 2014), 31. (Adams 2003)

* One individual or group immobilised during testing

Another difficulty in scoring aggression between individuals is the influence of observer bias. In aggression tests where observers are blind to the ants colonies of origin, studies are significantly less likely to find intra-colony aggression than in studies where the observer knows the origins of the ants (van Wilgenburg and Elgar 2013). These difficulties highlight the importance of a carefully considered experimental design and understanding of species attributes before using aggression to identify colony boundaries.

### Clustering

Another method which is often used to infer polydomy from the sharing of space is assessment of the distribution of nests in the environment. Territorial competition between nests means that in the absence of confounding factors, monodomous colonies are predicted to be equally spaced (overdispersed) through the environment (Levings and Traniello 1981). Deviation from this overdispersed pattern (clustering) suggests a shared space and certain level of mutual tolerance, and on this basis they can be viewed as members of the same multi-nest colony. This approach has been used in studies involving a variety of species to conclude that the nests are part of the same polydomous colony (Sudd et al. 1977; Dillier and Wehner 2004; Santini et al. 2011). In some populations, it has been found that at the local scale nests are clustered into ‘colonies’, but at the broader environmental scale these clusters of nests are overdispersed (Sudd et al. 1977; Dillier and Wehner 2004). The overdispersion of clusters may suggest that they are in competition with each other, adding support to the view that each cluster represents a socially connected multi-nest colony.

### Limitations

The observation of overdispersed clusters highlights the importance of scale when assessing dispersion of nests through the environment. Different studies have implemented this cluster-measuring approach at various environmental scales, such as the landscape (Sudd et al. 1977), the population (Dillier and Wehner 2004; Santini et al. 2011), and the within-colony scale (Pamilo et al. 1985; Cook et al. 2014). It is important to note that clustering at different spatial scales can reveal different information about the nests and colonies in question. For example, at the population level clustering may suggest mutual tolerance and membership of a polydomous colony (subject to the confounding factors discussed below). However, at the smaller, within-colony, scale it may be more likely to reveal information about the pattern of nest foundation within that colony. Many ant species reproduce by budding. During budding, workers and a queen (or queens) leave the nest on foot to found a new nest. Reproduction by budding will necessarily lead to spatial clustering, in this case clusters of nests will represent a history of repeated budding from an original founder nest, which may or may not also represent a selection of socially connected nests. The relevant scale is an important consideration when using clustering to infer membership of a polydomous colony and will depend on the ecology of the species being studied and the question being asked in the study.

Another factor which may confound an assessment of polydomy by clustering is nest density. In several species, populations with a higher nest density have been shown to have a higher degree of polydomy than populations with a lower nest density (Banschbach and Herbers 1996a; Bernasconi et al. 2005; Buczkowski 2011; Cao 2013). A link between nest density and polydomy makes judging the effect of clustering more difficult, as spatial proximity and social structure are no longer independent. Another potentially confounding factor when analysing clustering in space is environmental heterogeneity. For example, edge specialist species will tend to be found grouped together along the outside of a habitat, confounding the assessment of clustering (Sudd et al. 1977; Procter et al. 2015). A detailed understanding of species ecology is necessary to separate the linked effects of environment, optimal territorial strategy and dispersion (Hölldobler and Lumsden 1980; Levings and Traniello 1981). This is especially important in circumstances where the environment is changing; indeed, it has been suggested that polydomy is a strategy adopted by some species in response to either short (Banschbach and Herbers 1996a; Heller and Gordon 2006; Buczkowski and Bennett 2008) or long-term (Domisch et al. 2005; Denis et al. 2006; Sorvari and Hakkarainen 2007) changes in the environment. A thorough assessment of spatial clustering requires the necessary species- or population-specific knowledge to allow these factors to be accounted for in the analysis.

### Methods

Clustering is defined as nests in a group occurring closer together than would be expected if they were distributed randomly throughout the environment (Levings and Traniello 1981; Cook et al. 2014). Testing for spatial clustering, therefore, involves assessing whether points (in this case nests) differ in their spatial distribution from complete randomness (Table 1). One way to do this is to construct a null distribution by randomly spacing points in the environment. Metrics based on the distance between points (e.g. L and K functions (Ripley 1976)) can then be compared between the null and observed distributions (Cook et al. 2014). A significant difference between the distributions implies a significant difference from spatial randomness, i.e. overdispersion or clustering. These methods can give a quantitative assessment of the clustering of nests in the environment, but rely on a null model of complete spatial randomness, which may not always be appropriate. The scale at which the clustering is observed, (e.g. whole landscape or local area) will determine whether this method is informative as to the occurrence of polydomy in general or to find the specific boundaries of individual colonies.

### Sharing space: conclusions

Both aggression tests and clustering analysis can be used to assess the presence of polydomy when using the sharing of space to infer a social connection, as long as the ecology of the species is considered. Determining the limits of a polydomous colony using space has the advantage of potentially being quicker, simpler and cheaper than methods based on resource exchange or genetics. The disadvantage of this approach is that it does not reveal information about the functioning of the colonies or relatedness within and between them. This may not be a significant disadvantage when studying colonies at a broad population or landscape scale, where the exact extent of a colony may matter less than the more general level of cooperation and relatedness. In studies of invasive species, for example, it is often the case that resource exchange and relatedness are of less interest than the number and pattern of invasions. Shared-space methods of colony delimitation are therefore common in the study of invasive species such as the Argentine ant *Linepithema humile* (Buczkowski and Silverman 2006; Vogel et al. 2009; Vogel et al. 2010), the yellow crazy ant *Anoplolepis gracilipes* (Hoffmann 2014) and the invasive garden ant *Lasius neglectus* (Ugelvig et al. 2008). The extent to which these large invasive polydomous/supercolonial systems can be considered a single colony is beyond the scope of this review (but see Helanterä et al. 2009; Moffett 2012; Kennedy et al. 2014; Helanterä 2016).

Overall, it is clear that as long as the ecology of the study species is considered, the sharing of space can be a useful definition of polydomy, particularly due to its simplicity and lack of expense. However, as with other methods of assessing polydomy, it is important to be aware of the limitations of using shared space to define colony boundaries, and to be sure that this is a good definition to answer to biological question being considered.

## Sharing genes

Genetic tools allow inference of both evolutionary and historic patterns within and between populations of polydomous colonies. The evolution of supercoloniality in the Argentine ant, *Linepithema humile,* has been elucidated from genetic methods; it is suggested that the loss of variation in recognition cues following introduction to new areas has allowed supercolony formation (Giraud et al. 2002). The identification of source populations of invasive species is an example of the utility of genetic methods for identification of historical patterns, for example, the invasive garden ant, *Lasius neglectus*, is suspected to have expanded across Europe following small initial populations introduced from its native range (Ugelvig et al. 2008). Historical and evolutionary perspectives can complement more functional resource-based and spatial methods, potentially explaining why neighbouring nests are cooperating or clustered together.

### Theoretical basis

A colony is expected to be a genetic unit. By a genetic unit, we mean a group of individuals who are more related to one another than they are to individuals from the rest of the population. Greater genetic similarity to colony-mates as opposed to non-colony-mates is evidence of shared descent. Genetic divisions can be drawn on a number of measures, such as genetic relatedness, genetic differentiation or distinct matrilines, all of which will be discussed here.

Genetic delineation of colony boundaries offers a fundamentally different perspective on the colony as a unit compared to other methods discussed within this review. Here, we define a polydomous colony as per the definition of Debout et al. (2007), whereby spatially separate but socially connected nests are considered part of the same colony. Using this definition of a colony but drawing boundaries by means of genetic measures implicitly assumes that social connections form along genetic lines. Social connections appear to represent cooperative interactions (Buczkowski 2012; Gordon and Heller 2014; Ellis et al. 2014), and cooperation is more likely when the organisms in question are more related to one another (Hamilton 1964). It is therefore a reasonable assumption that social connections correlate with genetic links. However, while social connections can form along genetic lines (Banschbach and Herbers 1996b), genetic differentiation can be found within socially connected nest networks (Chapuisat et al. 1997; Holzer et al. 2009). Further, pairs of socially connected nests can also display no genetic distinction from nearby unconnected nests (Procter et al. 2016). Therefore, genetic methods for colony delineation may not correlate with social connections. It may be useful to use other, more functional, methods of colony delineation alongside genetic methods, to better understand the study system.

### Limitations

When designing a study to assess genetic differences between colonies, three factors affect the levels of sampling that is required: (i) the genetic variability of the colonies in question, (ii) the variability of the markers used, and (iii) the expected difference that separates one colony from another.

Workers within monogynous colonies, whether monodomous or polydomous, are highly related, and within-nest genetic diversity is fairly low, making distinguishing colony boundaries simple using genetic tools (Foitzik and Herbers 2001; Debout et al. 2003). In contrast in polygynous colonies, as the number of queens per colony increases so does the amount of genetic diversity contained within that colony, and worker relatedness decreases (Ross 2001), frequently approaching zero (Pedersen and Boomsma 1999; Tsutsui and Case 2001; Pamilo et al. 2005). As genetic diversity increases and worker relatedness decreases, the level of sampling must increase to detect genetic differences. Increasing the level of sampling can be done by sampling more workers per nest, assaying more loci per worker, utilising more variable loci, or a mixture of all three (Pedersen and Boomsma 1999). Practically, the high sampling effort required for highly polygynous systems means that genetic determination of colony structure can be expensive both in time and money compared to more ecological methods. Whether this investment is worthwhile will depend on the goals of the study in question.

Polydomous populations often exhibit short distance dispersal, leading to strong spatial genetic structuring (Sundström et al. 2005). This means that nests closer to one another are more genetically similar than to the rest of the population. Spatial genetic structuring needs to be accounted for in analyses before trying to distinguish between neighbouring colonies. The stronger the spatial structuring, the more of the variation in allele frequencies is explained by space, and not colony membership. In practice, this means that in a population with strong spatial genetic structuring, more loci or more variable loci are required to distinguish between neighbouring colonies.

Genetic differences build up over long timescales when the driver is mutation, rather than extreme events such as founder effects. The slow accumulation of genetic differences often allows inference of what has happened previously within or between populations, e.g. recent interbreeding of historically isolated populations of *Formica aquilonia* in response to forest cover change (Vanhala et al. 2014), or discovery of the sources of invasive populations of *Linepithema humile* (Tsutsui et al. 2001) and *Lasius neglectus* (Ugelvig et al. 2008). The long timescales necessary for differentiation to build up can also cause a problem because recently separated colonies may not yet have begun to diverge. As a result, neighbouring colonies may display clear ecological separation, but be indistinguishable in genetic terms (Procter et al. 2016). A combination of genetic methods with ecological or behavioural methods may allow clearer inference of colony boundaries.

Whereas net resource flow between nests may be directional, genetic measures are not. There is only a single measure of genetic differentiation or inter-nest genetic relatedness for a pair of nests; therefore, directionality is not possible in relatedness or differentiation. A colony, defined along social connections, would be expected to contain genetic variation, and not have identical allele frequencies in each nest. Therefore, it is possible that there will be situations where, in a group of three nests, nest A is not significantly differentiated from nest B or C, yet nests B and C show significant differentiation from one another. In this situation, it would be very difficult to know where to draw a colony boundary. We are unaware of any examples of this yet discovered, but a similar situation has been observed with aggression assays (Ugelvig et al. 2008).

In polygynous populations, polydomy has often been inferred from the presence of associated features of polydomy such as low relatedness of nestmates, the presence of budding dispersal and strong spatial genetic structuring of populations (Pamilo et al. 2005; Zinck et al. 2007). However, associated features do not indicate the scale of polydomous colonies, i.e. are the polydomous colonies two connected nests over 5 m, or 30 connected nests over 200 m? Furthermore, features associated with polydomy do not indicate the frequency of polydomy within the population, i.e. are all the colonies polydomous or is there a mix of monodomous and polydomous colonies? Inferences of polydomy from correlated traits are usually side effects of studies looking at other questions. However, the presence of polydomy can lead to false inference. For example, if multiple nests are sampled they are often assumed to be independent; the presence of polydomy within the population may mean that some of those sampled nests are, in fact, not independent data points. Analyses that do not take polydomous population structure into account may risk drawing incorrect conclusions (Seppä and Walin 1996).

### Methods

There are a range of methods which have been used to determine the genetic boundaries of multi-nest colonies (Table 1). Each method uses a different metric to assess which nests are genetically closest to one another. Perhaps the most obvious method to assess colony boundaries is genetic relatedness. Individuals within a colony should be more genetically related to one another than they are to individuals from other colonies within the population. To determine whether two nests are within the same polydomous colony, pairwise inter-nest relatedness estimates between workers of the nests in question can be examined. Expected inter-nest relatedness within the polydomous colony will depend on the level of relatedness found within each nest. Pairwise inter-nest relatedness estimates can then be adjusted to account for within-nest relatedness (Pedersen and Boomsma 1999), or the distribution of pairwise relatedness estimates can be compared to both within-nest relatedness and relatedness between distant unrelated nest pairs (Pamminger et al. 2014). Neither method has been widely applied, possibly because variation in pairwise relatedness estimates is high within samples. Therefore, discrimination would be difficult in situations with low within-nest relatedness, as is common in ants.

Instead of using relatedness to determine the degree of similarity between workers from separate nests, measures of genetic differentiation such as *F*_ST_ can be used to determine how different they are. Under this methodology, two nests that do not display statistically significant differentiation are said to be from the same colony, and nests that do display significant differentiation are said to be from different colonies (Elias et al. 2004; Dronnet et al. 2005; Steinmeyer et al. 2012). An alternative approach to *F*-statistics is *G*-distance (Pedersen and Boomsma 1999). This adapted measure of standard *G*-statistics (Sokal and Rohlf 1981) compares the heterogeneity of genotypes of workers sampled from different nests. The application of *G*-distance will produce a statistic whose magnitude correlates with genetic distance. The values for *G*-distance will be influenced by the number and variability of loci used, and therefore cannot be compared between studies. Furthermore, *G*-distance should be used to reinforce conclusions based on other genetic methods, not as a stand-alone method (Pedersen and Boomsma 1999). Conclusions about colony structure based on genetic differentiation should be made with care. This is especially true in polygynous species or populations where within-nest genetic diversity is high and in species with local dispersal where strong spatial genetic structuring is present. A lack of significant genetic differentiation is evidence of two nests being part of the same colony only if the study involves sufficiently numerous and variable loci to enable discrimination between neighbouring colonies. It is advisable to use statistical power analyses before embarking on studies dependent on genetic differentiation, and reinforcing conclusions based on genetic differentiation with other measures is recommended (Pedersen and Boomsma 1999; Dronnet et al. 2005).

Groupings of genetic data can be determined by Bayesian clustering algorithms, such as Structure (Pritchard et al. 2000), BAPS (Corander et al. 2003) or Geneland (Guillot et al. 2012), which are used widely in population level studies. These methods assess the number of clusters that best explain variation present in genetic data and the likelihood that each sampled individual belongs to each cluster. Clustering methods have been successfully applied to a range of polydomous species *Myrmica rubra* (Huszár et al. 2014), *Pheidole megacephala* (Fournier et al. 2012), *Formica paralugubris* (Holzer et al. 2009), *Formica exsecta* (Seppä et al. 2012), several times identifying potential polydomous colonies within apparently supercolonial or unicolonial populations (Holzer et al. 2009; Seppä et al. 2012). Multiple clustering methods can even be applied to the same study, to reinforce the validity of results (Holzer et al. 2009). There should be some caution in the spatial scale of data analysed by these methods, however, because large populations may contain genetic subdivisions above the level of the colony which the clustering algorithms will identify, masking smaller scale colony boundaries. Clustering methods can first be used to identify the highest spatial scale of populations structuring, and then subsequently applied within the initial structure to identify smaller clusters of nests, which may be polydomous colonies (Fournier et al. 2012). The necessary spatial scale for application of these analyses will have to be determined for each study.

When dealing with highly variable markers and trying to assign nests to groups, it can be most informative to look at rare genotypes within the population and the nests which share them. Common genotypes can often be found within neighbouring nests by chance, however, alleles rare within the population, but present in two neighbouring nests, are unlikely to be shared by chance (Pedersen and Boomsma 1999). If ants within neighbouring nests share alleles that are so rare in the population that they would be expected to be found in only a single nest, this is termed a ‘rare genotype sisterhood’ (Pedersen and Boomsma 1999). If neighbouring nests belong to ‘rare genotype sisterhoods’, then it is likely that the ants within them share common descent and so it is more likely that they are from the same colony. However, the lack of a rare genotype sisterhood does not prove that two nests are not within the same colony; they just may not have a genotype rare-enough to fulfil the necessary criteria. As mentioned earlier in this section, genetic differentiation due to mutation works on a longer timescale than ecological or behavioural processes. Neighbouring colonies in a population may share common descent and so belong to rare genotype sisterhoods without currently functioning as single colonies. This could make inferences from rare allele methods such as ‘rare genotype sisterhoods’ unreliable, and therefore we would only recommend their use for the purpose of defining colony boundaries in conjunction with other methods if at all.

Most studies that attempt to determine colony boundaries have done so using either allozymes or microsatellite markers. Though perfectly valid, these techniques have been restricted to nuclear DNA. Many ant species are known to display sex-biased dispersal, with males usually dispersing further than females (Doums et al. 2002; Clémencet et al. 2005; Soare et al. 2014). The sequencing of mitochondrial DNA (mtDNA) may help to reveal distinctions between nests that nuclear DNA does not. If there is strong sex-biased dispersal within the population, there may be no difference between neighbouring colonies in nuclear DNA, but these colonies could contain different matrilines, with different mitochondrial haplotypes. The utility of mtDNA will depend on how variable it is within the study population; in a population containing very few mitochondrial haplotypes, mtDNA sequence is unlikely to further inform colony structure.

We are not aware of any examples of next generation sequence data having been applied to the question of colony boundaries. With ever-decreasing costs we hope this will be an option in the near future, and the massively increased power available using those techniques may help to deal with some of the problems that currently exist in distinguishing colony boundaries. For an overview of the potential of next generation sequencing see Nygaard and Wurm (2015).

### Sharing genes: conclusions

As with any form of experimental design, the appropriate genetic methods to be used for determining colony boundaries will depend on the system in question. With species or populations where queen numbers are low, genetic tools can put colony boundaries in an evolutionary perspective with relative ease. However, in polygynous species or populations, we would recommend the application of functional measures of colony boundaries in addition to multiple genetic measures, to put the genetic patterns into ecological context. We would also recommend the use of statistical power analyses before embarking on a project, to ensure that there is enough power to distinguish any boundaries that may be present. Genetic tools offer the potential to elucidate evolutionary and historic patterns that are not available from other methods, and are therefore potentially very useful—but not without weaknesses.

## Discussion

Throughout this review, we have adhered to the definition of a polydomous colony as a group of spatially separate but socially connected nests (Debout et al. 2007). There are many ways in which social connections can be measured, as can be seen by the variety of methods we describe. It is therefore essential that any study using the term ‘polydomy’ specifies how it defines a social connection and how such connections are identified. Methods based around sharing resources can provide clear evidence of cooperation and a functional benefit to being within the same colony. Methods based on shared space inform about potential cooperation zones around nests. Shared genes can delineate groups of nests with common ancestry or highly related groups, and inform about historic patterns within and between populations. However, each method also has limitations. Shared resource methods give excellent data on the functioning of a colony but it may be difficult to infer the reasons underlying cooperation, without including cuticular hydrocarbons, genetic methods, or having tracked the same nests over long time periods. Shared space and shared genes methods suffer from the difficulty of disentangling current patterns from historic processes when the population history is unknown. Methods based on shared space are party to a particularly long list of potential limitations (see Shared Space section), but despite this, all these methods can be valuable if applied appropriately. Multiple methods used together will give a fuller understanding of the social organisation present within a population.

Polydomous species are both ecologically and phylogenetically diverse (Debout et al. 2007), and probably under-reported. Therefore, there is not an easily identifiable subset of ant species for which researchers need to be aware of polydomous nesting strategies. Any study looking at social organisation or within-population variation in ants needs to take into account colony structure and assess the scale over which it may occur. A study that includes samples from multiple nests within a population and assumes nests are independent, without considering the colony structure within that population, runs the risk of drawing fallacious conclusions. Polydomous colony structure could have large effects on studies assessing nest-level life-history traits, such as polygyny or offspring sex ratio, because different nests within the colony may adopt different strategies.

It is clear that the dichotomous view of monodomy or polydomy underrepresents the complexity found in the field. Polydomous colonies can vary from two spatially separate nests (Frizzi et al. 2015) to entire unicolonial populations (Holzer et al. 2006). Moreover, colony organisation strategies vary within species; the level of polydomy can vary with season (Elias et al. 2004; Gordon and Heller 2014), geographically between populations (Huszár et al. 2014; Ellis and Robinson 2014), and within a single population (Ellis et al. 2014). Colony structure should be viewed as a continuum, from entirely monodomous species at one extreme, to highly polydomous or unicolonial species at the other, and with variation within and between species and populations expected.

One of the most important ways in which ant social organisation varies is in the expected number of queens. In monogynous species, discrimination of neighbouring colonies can be simple and clear using genetic and aggression-based methods, and multiple methods of delineating colony boundaries often correlate well with one another (Table 3). When nests contain higher numbers of queens, care must be taken to ensure that genetic methods have sufficient power to distinguish between colonies (Pedersen and Boomsma 1999). The ability of workers to recognise their nestmates may decrease as the relatedness within the colony decreases (Pirk et al. 2001). Reduction in the efficacy of nestmate recognition will make the measuring of cuticular hydrocarbon profiles and aggression bioassays less useful as a diagnostic tool. As far as we are aware, cuticular hydrocarbon profiles have not been used as a direct diagnostic tool in studies of polydomy, however, aggression is widely used (see Shared Space section). As the level of polygyny increases it is, therefore, more important to assess polydomous colony boundaries thoroughly, using whichever methods fits the study. Furthermore, the use of multiple methods can be useful to ensure that all colony boundaries are detected.

**Table 3:**
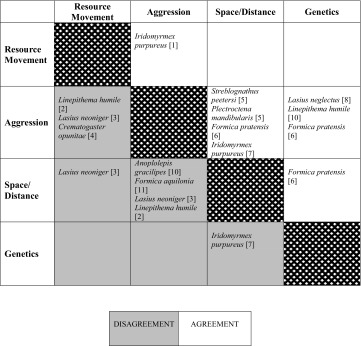
Studies comparing different methods of testing for polydomous boundaries

Each species entry shows an example where two methods of testing polydomous colony boundaries have been applied to the same population in the same study. Those in the top right (white squares) of the table show where results have agreed; those in the bottom left (grey) show where results have disagreed. References: 1. (Greenslade and Halliday 1983), 2. (Heller et al. 2008), 3. (Buczkowski 2012), 4. (Lanan and Bronstein 2013), 5. (Tanner and Keller 2012), 6. (Pirk et al. 2001), 7. (van Wilgenburg 2007), 8. (Ugelvig et al. 2008), 9. (Vogel et al. 2009), 10. (Hoffmann 2014), 11. (Sorvari and Hakkarainen 2004)

The scale at which colonies are organised can be affected by the environment. Colonies may be more likely to establish and maintain nests near resources (Holway and Case 2000; Ellis and Robinson 2015) and polydomous colony networks are structured to facilitate efficient resource flow (Cook et al. 2014). Polydomous colony organisation may therefore be a response to the distribution of resources (Robinson 2014), meaning that the ecological situation surrounding an ant colony may affect the level of polydomy it displays. If the interactions between ants and the environment are to be properly understood, accurate delineation of colony boundaries is essential.

Selection acts at multiple levels within a social insect population (Bourke and Franks 1995). As a cooperative and reproductive unit, the colony is an integral level of selection within the population. Evolutionary studies that ignore colony structure risk drawing fallacious conclusions. For example, queen-worker conflict can be explained by inclusive fitness (Sundström et al. 1996), but if polydomous colony boundaries have not been assessed such effects could be masked by different strategies present in different nests within a colony.

Deciding which methods to apply to a system will inevitably end in a trade-off between the desired aim of the study, the social organisation of the study species, and the resources available. The aim of the study must be the driving factor. Studies that are focussed on cooperative interactions should assess methods based on shared resources; studies aimed at assessing competition may be better suited to assessing methods based on shared space; and studies hoping to infer evolutionary or historic patterns may wish to use methods based on shared genes. Combinations of methods can yield greater insight than one alone, and help to deal with the weaknesses of a single method. Multiple methods may or may not draw the same conclusions (Table 3, Fig. 1); however, even when results differ, the understanding of the polydomous system increases with the use of multiple measures.

Both ecological and evolutionary studies must be aware that their view of what constitutes a colony may not be matched by researchers from another field. The functional interactions that a resource-based or behavioural study may identify will not necessarily be replicated by an evolutionary study using genetic tools. This is not necessarily problematic, as long as researchers do not assume that delineation of a colony using one method automatically means that all methods will also show the same boundary. Conclusions must acknowledge the limitations to generalisation that the use of a specific methodology brings.

Polydomy is found in both ecologically dominant keystone species (van Wilgenburg and Elgar 2007; Schlüns et al. 2009; Ellis and Robinson 2014) and economically and ecologically damaging invasive species (Buczkowski and Krushelnycky 2012; Fournier et al. 2012; Gordon and Heller 2014; Hoffmann 2014). Polydomy is therefore associated with a variety of extremely successful species, suggesting there are strong benefits to be gained using polydomous nesting strategy. Possible benefits of polydomy include spreading risk between nests, increasing the efficiency of resource discovery and exploitation, increasing the size of the colony above the constraints of single nest site and or release from the inefficiency of a very large nest (Robinson 2014). It is unlikely that there is a general reason why polydomy is a successful strategy, due to the large variety of species that adopt it, but further research is required to understand both how polydomous colonies are organised and why this strategy has been selected for.

### Conclusion

Polydomy is found throughout the Formicidae (Debout et al. 2007); therefore, any researchers working on ant species must assess their study population for polydomous colony structure, if the study could be affected by social organisation. We have surveyed a variety of different methods for studying polydomous colony organisation. These are based on shared resources, shared space and shared genes, and each method has its own strengths and weaknesses. The most important step in deciding which method to apply is to carefully fit the method to both the research question, and the study species. Once an appropriate method has been decided upon, the experimental design must account for the known limitations of that method, and if possible, apply one or more complementary methods in addition.
